# Exact solution of cilia induced flow of a Jeffrey fluid in an inclined tube

**DOI:** 10.1186/s40064-016-3021-8

**Published:** 2016-08-22

**Authors:** K. Maqbool, S. Shaheen, A. B. Mann

**Affiliations:** 1 Department of Mathematics and Statistics, International Islamic University, Islamabad, 44000 Pakistan; 2 Department of Mathematical Sciences, Federal Urdu University of Arts, Science and Technology, Near Zero Point, G-7/1, Islamabad, 44000 Pakistan

**Keywords:** Cilia, Jeffrey fluid, MHD, Inclined tube

## Abstract

The present study investigated the cilia induced flow of MHD Jeffrey fluid through an inclined tube. This study is carried out under the assumptions of long wavelength and low Reynolds number approximations. Exact solutions for the velocity profile, pressure rise, pressure gradient, volume flow rate and stream function are obtained. Effects of pertinent physical parameters on the computational results are presented graphically.

## Background

Dutch light microscopist Antoni van Leeuwenhoek discovered cilia in 1675. However, the first comprehensive account of cilia in English language may be credited to Sharpey (1835) who gave the detailed description of ciliary motion in a wide variety of reproductive and respiratory systems of mammals. During the last two decades much progress has been achieved in understanding of ciliary structures. These complex organelles have been examined by a number of experimental techniques. It is noticed that the difference between eukaryotic cilia and flagella is not rigorous. Cilia and flagella are organelles that are primarily used for the transportation of the cell. They propel the cell by flicking back and forth. Cilia motion propels the fluid in which it is moving by its cyclical movements which are asymmetric (Sleigh et al. 1988).

Cilia are short and many per cell and they are reminiscent of hairs whereas flagella are longer and fewer per cell and are reminiscent of a tail. Cilia or flagella shows a variety of beating patterns depending on the surrounding geometry as described in literature (Lindemann and Lesich 2010; Ibacez-Tallon et al. 2003). Cilia beating has been used in regulation (locomotion, ingestion, mixing, etc.). In general, a two-stroke recovery-effective motion is displayed by a single cilium forming the beating pattern. When the cilium is moving towards itself and the neighboring boundary, the recovery (backward) stroke starts, reducing there by the drag on the neighboring fluid in the backward, whereas during the forward stroke, the cilium moves forward into the fluid, dragging the large possible volume of extended fluid. These beating patterns have been considered by various investigators in their works on Cilia motion (Brennen and Winet 1977; Verdugo 1982; Takahashi and Shingyoji 2002; Sanderson and Sleigh 1981; Lardner and Shack 1972).

During the combined motion of cilia, it seems to beat in an organized manner such that upper layer of cilia is deformed in a wave-like manner. This wave is known as metachronal wave, caused by a small angular difference between neighboring cilia, and are just like the waves formed by waving spectators in stadiums. The beat frequency and the metachronal wavelength of cilia are defined as *f* = *c*/2*π* and *λ* = 2*π*/*k* respectively where *c* is the wave speed and *k* is the wave number. This collective motion of cilia has been used in many biological studies (Takahashi and Shingyoji 2002; Murakami and Takahashi 1975).

According to the relationship between the forward stroke of the motile cilia and their dynamics, different types of metachronal waves are classified. When the direction of the forward stroke and direction of propagative metachronal wave is same the symplectic beat pattern is formed whereas an antiplectic beat coordination is formed if both directions are opposite to each other (Knight-Jones 1954; Blake 1972).

For the locomotion of mucus by cilia, shear-thinning and elastic effects are assumed. It can diminish the fluid flow opposition at the cilia layer and preserve a semi rigid plane at the superior mucus layer which facilitates the most favorable transport of particles in the respiratory tract. Because of the significance of mucus in an individual’s health, many attempts have been made to describe the tracheobronchial mucus transportation (Ross and Corrsin 1974; Blake 1975; Barton and Raynor 1967; Fulford and Blake 1986).

More recently Siddiqui et al. (2014a) examined the hydromagnetic cilia flow of a Newtonian fluid in a symmetric channel using long wavelength approximation. Numerous past experimental observations show that many biological fluids exhibit non-Newtonian characteristics (Siddiqui et al. 2015; Shukla et al. 1980; Srivastava et al. 1983; Brasseur et al. 1987; Narahari and Sreenadh 2010). It is important to note that the cilia induced fluid flow problems play a significant role in non-Newtonian fluid mechanics. Jeffrey fluid model is one of the simplest but the most extensively used models for rheological fluid transports (Hayat et al. 2010; Nadeem and Akbar 2009; Pandey and Tripathi 2010; Kavitha et al. 2012). Viscous properties of human sperm are experimentally found to exhibit Jeffrey behavior (Nadeem and Akram 2010a). Further Jeffrey fluid model has been studied in mechanism of fluid transport specifically in biological systems e.g., urine transport from kidney to bladder through ureter, movement of chime in the gastrointestinal tract, the movement of spermatozoa in the ducts efferent of the male reproductive tract, ovum in the female fallopian tube, the locomotion of some worms and transport of lymph in the lymphatic vessels (Hayat and Ali 2008). Since the biological fluids are electrically conducted in nature and cilia are present in the airways of lungs which help in protecting from inhaled dust, bacteria and other harmful substances by highly viscous and non-Newtonian mucous (Jeffrey fluid) mainly consisting of water, salt and glycocylated mucin protein (MHD sources) which show the significance of cilia induced flow of MHD Jeffrey fluid in the respiratory tract of humans. The cilia induced flow of Jeffrey fluid in respiratory tract helps in removal of harmful substances trapped in the mucous which are transported along the airways and out of the lungs by the activity of dense mat of microscopic cilia (Smith et al. 2008). By adjusting the various parameters involved in the present study may lead to obtaining results beneficial for removal of tracheobronchial mucous in the respiratory tract. In the present study, mucous is represented by the linearized Jeffrey model valid for small rates of shear and motion is generated due to constant pressure gradient produced by the cilium tips. Different studies for the Jeffrey fluid model in a symmetric channel under the effect of magnetic field are also available in Hayat and Ali (2008), Kothandapani and Srinivas (2008), Hayat et al. (2008), Nadeem and Akram (2010b), Nallapu and Radhakrishnamacharya (2015) and Saravana et al. (2011). The term cilia used in this paper is restricted to ciliated epithelium.

The aim of this work is to study the transport characteristics through the cilia induced MHD flow of Jeffrey fluid under the low Reynold’s number and long-wavelength approximation in an inclined tube.

## Problem formulation

**Fig. 1:**
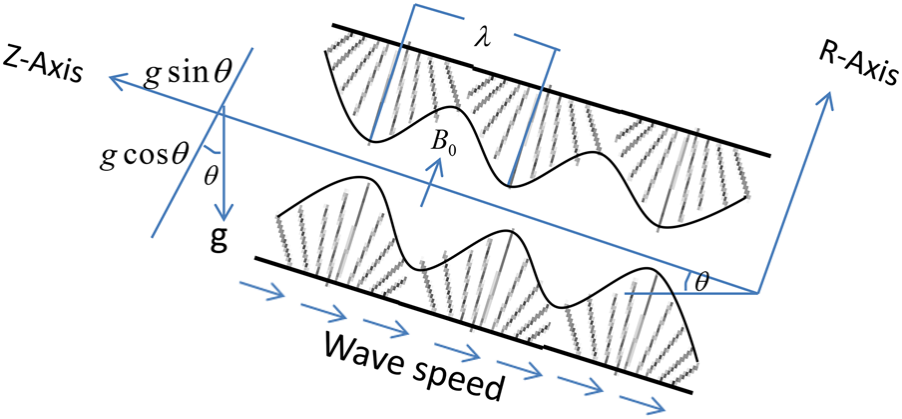
Geometry of the problem

Consider the fluid transport characteristics of an incompressible MHD Jeffrey fluid in an inclined tube under the action of ciliary beat that generates a metachronal wave. The tube having ciliated walls is large in length as compared to radius and a symplectic metachronal wave moves to the right with wave speed *c* (Siddiqui et al. 2014a, 2015; Smith et al. 2008). The cylinderical coordinate system $(R,\phi ,z)$ is appropriate for the above stated problem. The geometry of the problem under consideration is depicted in Fig. 1.

Cilia are present in the physical model as we have considered the ciliated tube and are hinged near the base at the inner walls of the tube and the fluid motion is generated due to their swing. In existing literature many researchers (Siddiqui et al. 2010, 2015; Akbar et al. 2014, 2016) considered the cilia tips moving in elliptical path which can be represented mathematically in the form

1 $$R=H=f(z,t)=a+a\epsilon \cos \left( \frac{2\pi }{\lambda }(Z-ct)\right),$$

which is the equation of extensible tube wall, where *a* is the mean radius of tube, $\epsilon$ is the non-dimensional parameter and *λ* and *c* are the wavelength and wave speed of metachronal wave respectively. The horizontal position of cilia tips can be written as

2 $$Z=g(Z,Z_{0},t)=Z_{0}+a\epsilon \alpha \sin \left( \frac{2\pi }{\lambda }(Z-ct)\right),$$

where $Z_{0}$ is the reference position of cilia and *α* is the eccentricity of ellipse. According to no slip condition the velocity of cilia tips and of fluid adjacent to cilia tips are same, so the *Z* and *R* components of velocity are given to be

3 $$W=\left. \frac{\partial Z}{\partial t}\right| _{_{Z=Z_{0}}}=\frac{\partial g}{\partial t}+\frac{\partial g}{\partial Z}\frac{\partial Z}{\partial t}=\frac{\partial g}{\partial t}+\frac{\partial g}{\partial Z}W$$

and

4 $$U=\left. \frac{\partial R}{\partial t}\right| _{Z=Z_{0}}=\frac{\partial f}{\partial t}+\frac{\partial f}{\partial Z}\frac{\partial Z}{\partial t}=\frac{\partial f}{\partial t}+\frac{\partial f}{\partial Z}W,$$

respectively. Using Eqs. (1–2) in Eqs. (3–4), we arrive at

5 $$W=\frac{-\left( \frac{2\pi }{\lambda }\right) \left[ \epsilon a\alpha c\cos \left( \frac{2\pi }{\lambda }\right) (Z-ct)\right] }{1-\left( \frac{2\pi }{\lambda }\right) \left[ \varepsilon a\alpha c\cos \left( \frac{2\pi }{\lambda }\right) (Z-ct)\right] } $$

and

6 $$U=\frac{\left( \frac{2\pi }{\lambda }\right) \left[ a\epsilon \alpha c\sin \left( \frac{2\pi }{\lambda }\right) (Z-ct)\right] }{1-\left( \frac{2\pi }{\lambda }\right) \left[ \epsilon a\alpha c\cos \left( \frac{2\pi }{\lambda }\right) (Z-ct)\right] },$$

where *U* and *W* are the velocity components in axial and radial directions, respectively. A uniform magnetic field of strength *B*_0_ is applied in the direction perpendicular to the cilia induced axial flow. The transformations relating the wave frame (*r*, *z*), moving with velocity *c*, to the fixed frame (*R*, *Z*) are given by

7 $$z=Z-ct,\quad r=R,\quad w=W-c,\quad u=U\quad \hbox{and}\quad p(z,r)=P(Z,R,T),$$

where *P* is the pressure in fixed frame.

The mass and momentum equations which govern the unsteady MHD flow of an incompressible Jeffrey fluid in an inclined tube can be written as (Hayat et al. 2008; Nadeem and Akram 2010b; Nallapu and Radhakrishnamacharya 2015)

8 $$\varvec{\nabla }.{\mathbf{V}}=0, $$

where

9 $${\mathbf{V}}=\left[ u(r,z),0,w(r,z)\right]$$

and

10 $$ \rho \frac{d{\mathbf{V}}}{dt}=\hbox{div}{\mathbf{T}}+{\mathbf{J}}\times {\mathbf{B}}+\rho {\mathbf{g}}, $$

where

11 $$ {\mathbf{T}}= -p{\mathbf{I}}+{\mathbf{S}}, $$

12 $$ \varvec{S}= \frac{\mu }{1+\lambda _{1}}\left( {\mathbf{A}}_{1}+\lambda _{2}\frac{d{\mathbf{A}}_{1}}{dt}\right) $$

and

13 $$\begin{aligned} \varvec{\nabla } \varvec{\cdot } {\mathbf{B}} &= 0,\ \varvec{\nabla } \times {\mathbf{B}}= \mu _{m}{\mathbf{J}},\quad \varvec{\nabla } \times {\mathbf{E}}= -\frac{\partial {\mathbf{B}}}{\partial t},  \\ {\mathbf{J}}&=  \sigma \left[ {\mathbf{E}}+{\mathbf{V}}\times {\mathbf{B}} \right] \end{aligned}$$

In above equations *ρ* is the fluid density, *σ* is the electrical conductivity, **g** is the acceleration force, **T** is the Cauchy stress tensor, **I** is the identity tensor, **S** is the extra stress tensor, **A**_1_ is the first Rivlin Ericksen tensor, $\lambda _{i}(i=1,2)$ are the fluid parameters, **J** is the current density, $\mu _{m}$ is the magnetic permeability and **E** is the total electric field, **B** is the total magnetic field so that ${\mathbf{B}}={\mathbf{B}}_{0}+{\mathbf{b}}$, **b** is the induced magnetic field and is neglected compared with applied magnetic field ${\mathbf{B}}_{0}$ which is in the transverse direction of the flow. In the present analysis, it is assumed that no applied and polarization voltages exist i.e. **E** = 0. This then corresponds to the case where no energy is added or extracted from the fluid by the electric field.

In the light of above assumptions and due to small magnetic Reynold’s number the magnetohydrodynamic force in Eq. (10) is given by

14 $$ {\mathbf{J}}\times {\mathbf{B}}=-\sigma B_{0}^{2}(w+c)\hat{e}_{z} $$

The following dimensionless variables are used to make the quantities dimensionless.

15 $$\begin{aligned} z^{*}&=  \frac{z}{\lambda }, \quad r^{*}=\frac{r}{a},\quad u^{*}=\frac{u}{\beta c},\quad w^{*}=\frac{w}{c},\quad h^{*}=\frac{H}{a},\quad p^{*}=\frac{a\beta }{c\mu }p, \quad t^{*}=\frac{ct}{\lambda },  \\ \beta&=  \frac{a}{\lambda },\quad S_{ij}^{*}=\frac{a}{\mu c}S_{ij},\quad M=\sqrt{\frac{\sigma }{\mu }}aB_{0},\quad \textit{Re}=\frac{\rho ca}{\mu },\quad {\text{and}} \quad Fr=\frac{c^{2}}{ga}. \end{aligned}$$

In view of (15), the non-dimensional radial and axial momentum equations become

16 $$ \textit{Re}\beta ^{3}\left[ \frac{\partial u}{\partial t}+u\frac{\partial u}{\partial r}+w\frac{\partial u}{\partial z}\right]= -\frac{\partial p}{\partial r}+\beta \frac{1}{r}\frac{\partial }{\partial r}(rS_{rr})-\beta \frac{S_{\theta \theta }}{r}+\beta ^{2}\frac{\partial S_{zr}}{\partial z}-\beta \frac{\textit{Re}}{\textit{Fr}}\cos \theta ,$$

17 $$ \beta \left[ \frac{\partial w}{\partial t}+u\frac{\partial w}{\partial r}+w\frac{\partial w}{\partial z}\right] =  -\frac{\partial p}{\partial z}+\frac{1}{r}\frac{\partial }{\partial r}(rS_{rz})+\beta \frac{\partial S_{zz}}{\partial z}-M^{2}(w+1)+\frac{\textit{Re}}{\textit{Fr}}\sin \theta ,$$

where

18 $$ S_{rz}=\frac{\mu }{1+\lambda _{1}}\left( \frac{\partial w}{\partial r}+\beta \frac{\partial u}{\partial z}\right) +\lambda _{2}\left( \beta u\frac{\partial ^{2}w}{\partial r^{2}}+u\beta ^{3}\frac{\partial ^{2}u}{\partial r\partial z}+\beta w\frac{\partial ^{2}w}{\partial r\partial z}+\beta ^{3}w\frac{\partial ^{2}u}{\partial z^{2}}\right) .$$

The appropriate boundary conditions will take the form

19 $$ u=\frac{2\pi \epsilon \sin (2\pi z)}{1-2\pi \epsilon \alpha \beta \cos (2\pi z)}$$

and

20 $$ w=\frac{-2\pi \epsilon \alpha \beta \cos (2\pi z)}{1-2\epsilon \pi \alpha \beta \cos (2\pi z)}-1, \quad  \hbox{at}\; r=h=1+\epsilon \cos (2\pi z). $$

Further, the centerline symmetry requires

21 $$ \frac{\partial w}{\partial r}=0,\quad\hbox{at}\,r=0. $$

Since the flows in small diameter tubules are inertia free therefore the analysis in the present study can be approximated under the long wavelength or small Reynold’s number approximations i.e., $(\beta \rightarrow 0,\, \textit{Re}\ll 1)$ (Shapiro et al. 1969). So the governing equations with boundary conditions (16–21) under the long wavelength approximation can be simplified to

22 $$\frac{\partial ^{2}w}{\partial r^{2}}+\frac{1}{r}\frac{\partial w}{\partial r}-M^{2}(1+\lambda _{1})w=\left( 1+\lambda _{1}\right) \left( \frac{\partial p}{\partial z}-\frac{\textit{Re}}{\textit{Fr}}\sin \theta +M^{2}\right) ,$$

23 $$\frac{\partial p}{\partial r}=0,$$

24 $$\frac{\partial w}{\partial r}=0,\quad\hbox{at}\; r=0\quad \hbox{and}\quad w=w(h)=-1-2\pi \epsilon \alpha \beta \cos (2\pi z)$$

and

25 $$ u=u(h)=2\pi \epsilon \sin (2\pi z)+\beta (2\pi \epsilon )^{2}\alpha \sin (2\pi z)\cos (2\pi z).$$

## Solution of the problem

From Eq. (23), it is evident that $p\ne p(r)$ and hence *p* is the function of *z* alone therefore Eq. (22) becomes

26 $$ \gamma ^{2}\frac{\partial ^{2}w}{\partial r^{2}}+\gamma \frac{\partial w}{\partial r}-M^{2}\gamma ^{2}(1+\lambda _{1})w=(1+\lambda _{1})\gamma ^{2}\left( \frac{dp}{dz}-\frac{\textit{Re}}{\textit{Fr}}\sin \theta +M^{2}\right) , $$

with boundary conditions

27 $$ u=u(h)=2\pi \varepsilon \sin (2\pi z)+\beta (2\pi \varepsilon )^{2}\alpha \sin (2\pi z)\cos (2\pi z), $$

and

28 $$ \frac{\partial w}{\partial r}=0,\quad \hbox{at} \; r=0\quad\hbox{and}\quad w=w(h)=-1-2\pi \varepsilon \alpha \beta \cos (2\pi z). $$

Solving the non-homogeneous Bessel’s Eq. (26) with the help of boundary conditions (27, 28), we arrive at

29 $$ w=w(h)\frac{I_{0}(\sqrt{1+\lambda _{1}}Mr)}{I_{0}(\sqrt{1+\lambda _{1}}Mh)}+\left( 1+\frac{1}{M^{2}}\frac{dp}{dz}-\frac{1}{M^{2}}\frac{\textit{Re}}{\textit{Fr}}\sin \theta \right) \left( \frac{I_{0}(\sqrt{1+\lambda _{1}}Mr)}{I_{0}(\sqrt{1+\lambda _{1}}Mh)}-1\right) , $$

where $I_{0}$ is the modified Bessel’s function of first kind and order zero.

Integrating Eq. (8)

30 $$ \int \limits _{0}^{h}\left[ \frac{1}{r}\frac{\partial (ru)}{\partial r}+\frac{\partial w}{\partial z}\right] rdr=0,$$

we yield

31 $$ hu(h)+\frac{1}{2}\frac{\partial q}{\partial z}-h\frac{\partial h}{\partial z}w(h)=0, $$

where $q=2\int \nolimits _{0}^{h}wrdr$. Equation (31) can be written as

32 $$ \frac{\partial q}{\partial z}=2h\left( \frac{\partial h}{\partial z}w(h)-u(h)\right) =0.$$

Again integrating Eq. (29) over the cross section of tube, the pressure gradient can be written in terms of volume flow rate as

33 $$\begin{aligned} \frac{dp}{dz}&=  \frac{q\sqrt{1+\lambda _{1}}M^{3}I_{0}(\sqrt{1+\lambda _{1}}Mh)-2M^{2}hw(h)I_{1}(\sqrt{1+\lambda _{1}}Mh)}{2hI_{1}(\sqrt{1+\lambda _{1}}Mh)-h^{2}M\sqrt{1+\lambda _{1}}I_{0}(\sqrt{1+\lambda }Mh)}  \\&\quad+M^{2}\frac{\textit{Re}}{\textit{Fr}}\sin \theta -M^{2}, \end{aligned}$$

where $I_{1}$ is the modified Bessel’s function of first kind and order one. Substituting the value of $\frac{dp}{dz}$ in Eq. (29), we get

34 $$\begin{aligned} w&=  w\left( h\right) \frac{I_{0}\left( \sqrt{1+\lambda _{1}}Mr\right) }{I_{0}\left( \sqrt{1+\lambda _{1}}Mh\right) }+\left( \frac{I_{0}(\sqrt{1+\lambda _{1}}Mr)}{I_{0}(\sqrt{1+\lambda _{1}}Mh)}-1\right)  \\&\times \left\{ 1+\left( \frac{q\sqrt{1+\lambda _{1}}MI_{0}(\sqrt{1+\lambda _{1}}Mh)-2hw(h)I_{1}(\sqrt{1+\lambda }Mh)}{2hI_{1}(\sqrt{1+\lambda _{1}}Mh)-h^{2}M\sqrt{1+\lambda _{1}}I_{0}(\sqrt{1+\lambda _{1}}Mh)}\right. \right.  \\&\left. \left. +M^{2}\frac{\textit{Re}}{\textit{Fr}}\sin \theta -M^{2}\right) -\frac{1}{M^{2}}\frac{\textit{Re}}{\textit{Fr}}\sin \theta \right\} . \end{aligned}$$

In order to find the pressure rise, we shall integrate Eq. (33) with respect to *z* from 0 to *h* which results in

35 $$ \Delta p=qM^{3}\sqrt{1+\lambda }I_{1}^{*}-2M^{2}I_{2}^{*}+M^{2}\frac{\textit{Re}}{\textit{Fr}}\sin \theta -M^{2},$$

where

36 $$ I_{1}^{*}=\int \limits _{0}^{1}\frac{I_{0}\left( \sqrt{1+\lambda }Mh\right) }{2hI_{1}\left( \sqrt{1+\lambda }Mh\right) -h^{2}M\sqrt{1+\lambda }I_{0}\left( \sqrt{1+\lambda }Mh\right) }dz,$$

and

37 $$ I_{2}^{*}=\int \limits _{0}^{1}\frac{hw(h)I_{1}\left( \sqrt{1+\lambda }Mh\right) }{2hI_{1}\left( \sqrt{1+\lambda }Mh\right) -h^{2}M\sqrt{1+\lambda }I_{0}\left( \sqrt{1+\lambda }Mh\right) }dz.$$

The integrals appearing in (36–37) are solved by using the software “**Mathematica**”. The relation between *q* and dimensionless volume flow rate *Q* is given by

38 $$ Q=2\int \limits _{0}^{h}WRdR=2\int \limits _{0}^{h}\left( w+1\right) rdr=q+h^{2}.$$

The mean volume flow rate for the time period $T=\frac{\lambda }{c}$ is

39 $$ \bar{Q}=\frac{1}{T}\int \limits _{0}^{T}Qdt^{*}=q+1+0.5\epsilon ^{2}.$$

Now using the relation

40 $$ \Delta p=(\bar{Q}-1-0.5\epsilon ^{2})M^{3}\sqrt{1+\lambda }I_{1}^{*}-2M^{2}I_{2}^{*}+M^{2}\frac{\textit{Re}}{\textit{Fr}}\sin \theta -M^{2}, $$

Equation (40) can be written as

41 $$ \bar{Q}=\frac{\Delta p+M^{2}-M^{2}\frac{\textit{Re}}{\textit{Fr}}\sin \theta +2M^{2}I_{2}^{*}}{2I_{1}^{*}M^{3}\sqrt{1+\lambda }}+1.$$

The radial velocity for the case of Jeffrey fluid can be evaluated from Eq. (30) as

42 $$\begin{aligned} u&=  -\frac{\partial w\left( h\right) }{\partial z}\frac{I_{1}\left( \sqrt{1+\lambda _{1}}Mr\right) }{I_{0}\left( \sqrt{1+\lambda _{1}}Mh\right) }+w\left( h\right) \frac{I_{1}\left( \sqrt{1+\lambda _{1}}Mr\right) I_{1}\left( \sqrt{1+\lambda _{1}}Mh\right) }{I_{0}^{2}\left( \sqrt{1+\lambda _{1}}Mh\right) }\frac{\partial h}{\partial z}  \\&\quad+\left( \frac{1}{M^{2}}\frac{dp}{dz}+1-\frac{1}{M^{2}}\frac{\textit{Re}}{\textit{Fr}}\sin \theta \right) \left( \frac{I_{1}(\sqrt{1+\lambda _{1}}Mr)I_{1}(\sqrt{1+\lambda _{1}}Mh)}{I_{0}^{2}(\sqrt{1+\lambda _{1}}Mh)}\frac{\partial h}{\partial z}\right)  \\&\quad-\frac{1}{M^{2}}\frac{d^{2}p}{dz^{2}}\left( \frac{I_{1}(\sqrt{1+\lambda _{1}}Mr)}{M\sqrt{1+\lambda _{1}}I_{0}(\sqrt{1+\lambda _{1}}Mh)}-\frac{r}{2}\right) . \end{aligned}$$

Similarly, the stream function can be evaluated by the help of velocity profile and is given by

43 $$\begin{aligned} \psi&=  rw(h)\frac{I_{1}(\sqrt{1+\lambda _{1}}Mr)}{I_{0}(\sqrt{1+\lambda _{1}}Mh)}+\left( \frac{1}{M^{2}}\frac{dp}{dz}+1-\frac{1}{M^{2}}\frac{\textit{Re}}{\textit{Fr}}\sin \theta \right)  \\&\quad \times \left( \frac{rI_{1}(\sqrt{1+\lambda _{1}}Mr)}{M\sqrt{1+\lambda _{1}}I_{0}(\sqrt{1+\lambda _{1}}Mh)}-\frac{r^{2}}{2}\right) . \end{aligned}$$

The results presented above correspond to the results of Siddiqui et al. (2014b) when both *λ*_1_ and inclination angle *θ* approach zero.

## Numerical results and discussion

In this section, the graphical illustrations of the results obtained in the last sections are presented to show the effects of various parameters of interest. In particular the effect of distinct parameters for the pressure rise, axial velocity, axial pressure gradient and stream function are observed.

**Fig. 2:**
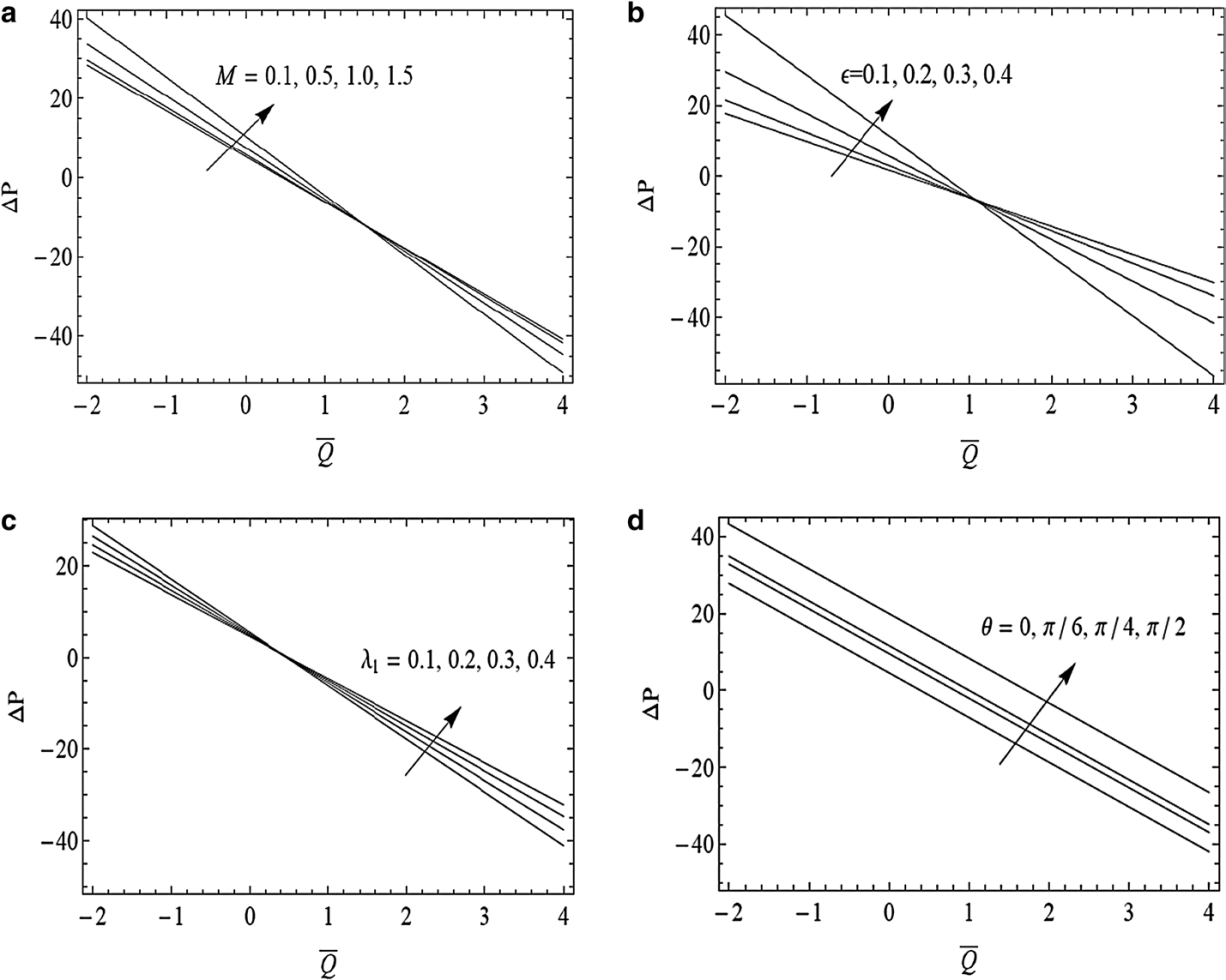
Variation of pressure rise for Δ*p* with time average flux $\bar{Q}$ for $\alpha =0.4, \beta =0.4,Fr=0.1$ and $\textit{Re}=0.1$ with **a** $\epsilon =0.3,\lambda _{1}=0.1,\theta =\frac{\pi }{3}$ and $M=0.1,0.5,1,1.5$, **b** $M=0.3,\lambda _{1}=0.1,\theta =\frac{\pi }{3}$ and $\epsilon =0.1,0.2,0.3,0.4$, **c** $\epsilon =0.3,M=0.1,\theta =\frac{\pi }{3}$ and *λ*_1_ = 0.1, 0.2, 0.3, 0.4, **d** $\epsilon =0.3,M=0.3,\lambda _{1}=0.1, \theta =0,\frac{\pi }{6}, \frac{\pi }{4},\frac{\pi }{2}$

The variation of pressure rise Δ*p* with time average flux $\bar{Q}$, for different values of Hartmann number *M*, cilia length $\epsilon$, Jeffrey parameter *λ*_1_, and angle of inclination *θ*, are examined in Fig. 2a–d. It is noted that pressure rise increases with an increase in Hartmann number *M* in the region $(\bar{Q}<1$, $\Delta p>0)$. However a converse trend is noted in region $(\bar{Q}>1$, $\Delta p<0)$. Figure 2b shows an increase in pressure rise with increase in $\epsilon$ in the interval $-2\le \bar{Q}\le 1$. However, it decreases by increasing $\epsilon$ for $\bar{Q}>1$. Figure 2c indicates a decrease in pressure rise by increasing Jeffrey parameter *λ*_1_ in the interval $-2\le \bar{Q}\le 0.5$ while its behavior is reversed when $0.5\le \bar{Q}\le 4$. Figure 2d indicates that pressure rise increases by increasing angle of inclination *θ* in the whole interval $-2\le \bar{Q}\le 4$.

**Fig. 3:**
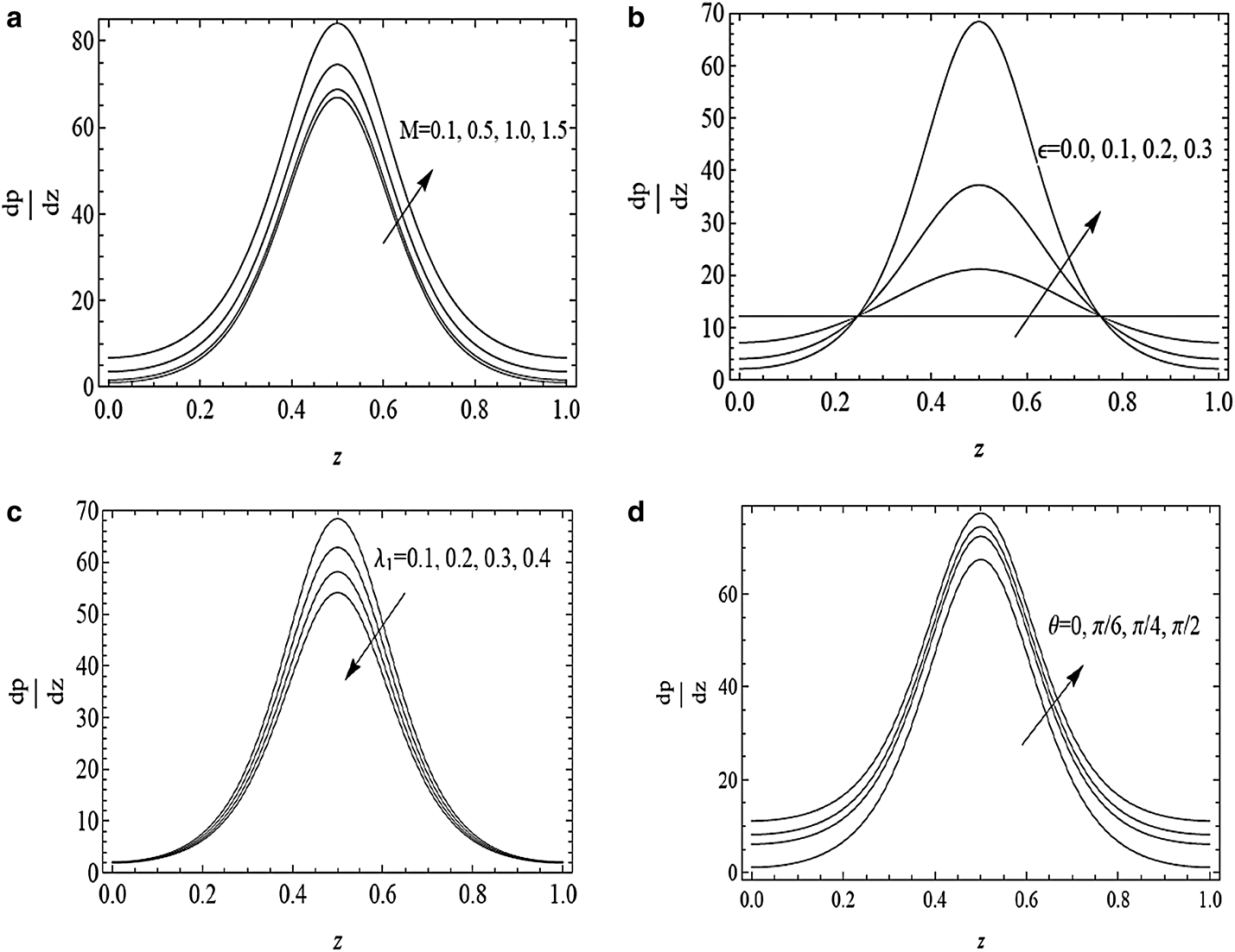
Variation of axial pressure gradient $\frac{dp}{dz}$ with *z* for $\alpha =0.4,\beta =0.4,Fr=0.1$ and $\textit{Re}=0.1$ with **a** $\epsilon =0.3,\lambda _{1}=0.1, \theta =\frac{\pi }{3}$ and $M=0.1,0.5,1,1.5$, **b** $M=0.3,\lambda _{1}=0.1,\theta =\frac{\pi }{3}$ and $\epsilon =0.0,0.1,0.2,0.3$, **c** $\epsilon =0.3,M=0.3,\theta =\frac{\pi }{3}$ and *λ*_1_ = 0.1, 0.2, 0.3, 0.4, **d** $\epsilon =0.3,M=0.3,\lambda _{1}=0.1, \theta =0,\frac{\pi }{6},\frac{\pi }{4},\frac{\pi }{2}$

The variation of axial pressure gradient $\frac{dp}{dz}$ with *z* for different values of $M,\epsilon ,\lambda _{1}$ and *θ* is depicted in Fig. 3a–d. It is observed that pressure gradient is small in the region $0\le z\le 0.28$ and $0.78\le z\le 1$ and fluid can flow easily without applying a large pressure gradient but a large amount of pressure gradient is required to maintain the same flux in the region $0.28<z<0.78$. Also by increasing the Hartmann number *M*, the amplitude of $\frac{dp}{dz}$ increases. Fig. 3b reveals that the amplitude of the pressure gradient increases with an increase of cilia parameter $\epsilon$ in the region $0.28\le z\le 0.78$. From Fig. 3c it is noticed that by increasing the Jeffrey parameter *λ*_1_, the amplitude of the pressure gradient in the centre of the tube decreases whereas no pressure gradient is required to maintain the flow at the ends of the tube. Figure 3d shows that increase in angle of inclination increases the amplitude of the pressure gradient i.e., inclined tube requires a large amount of pressure gradient to flow in the region [0.28, 0.78] as compared to the inlet and outlet of the tube.

**Fig. 4:**
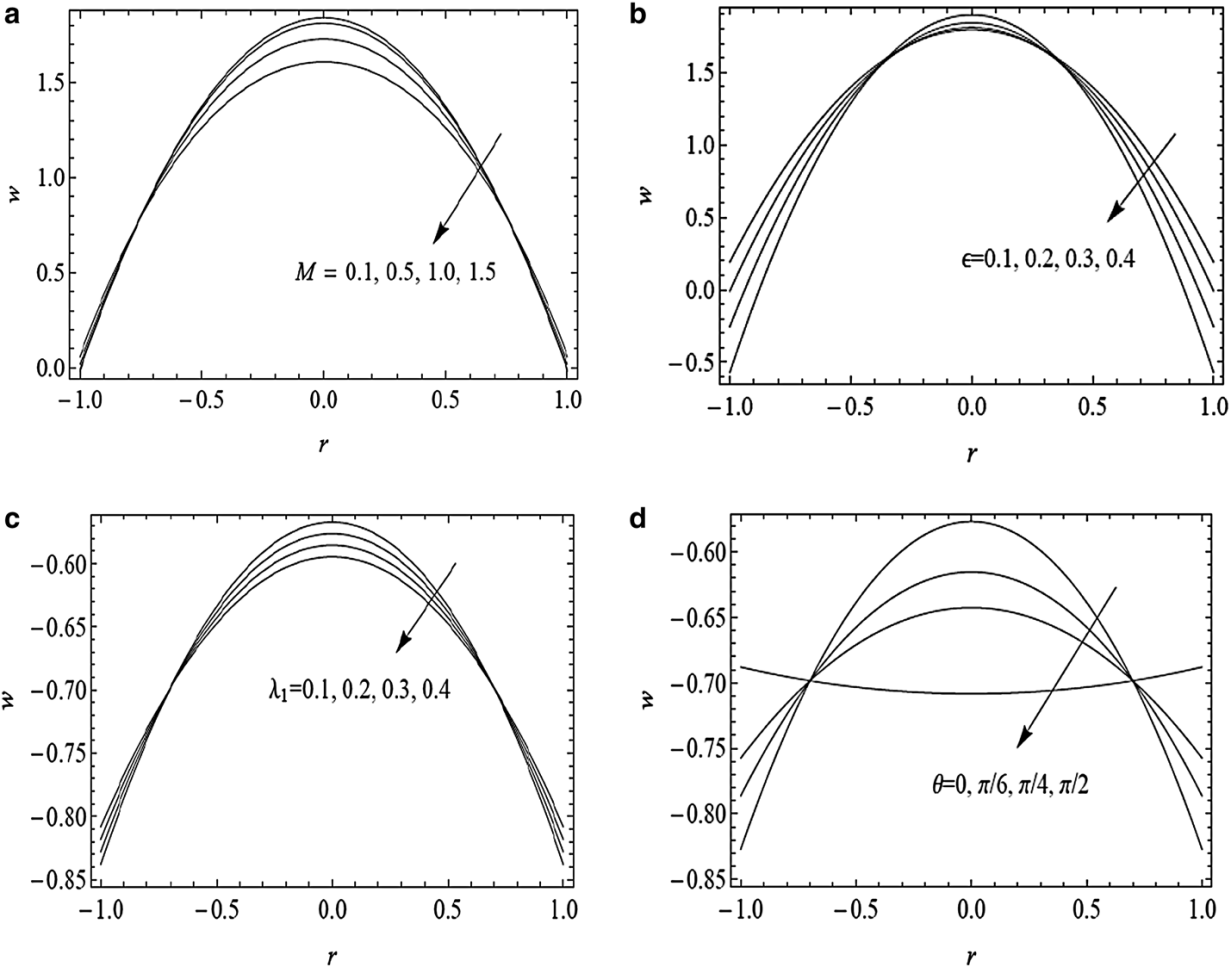
Variation of axial velocity *w* with *r* for $\alpha =0.4, \beta =0.4, Fr=0.1, \bar{Q}=1.5$ and *z* = 1 with **a** $\epsilon =0.3, \lambda _{1}=0.1, \theta = \frac{\pi }{3}$ and $M=0.1, 0.5, 1, 1.5$, **b** $M=0.3, \lambda _{1}=0.1, \theta =\frac{\pi }{3}$ and $\epsilon =0.0, 0.1, 0.2, 0.3$, **c** $\epsilon =0.3, M=0.3, \theta =\frac{\pi }{3}$ and *λ*_1_ = 0.1, 0.2, 0.3, 0.4, **d** $\epsilon =0.3, M=0.3, \lambda _{1}=0.1, \theta =0, \frac{\pi }{6}, \frac{\pi }{4}, \frac{\pi }{2}$

In Fig. 4a–d axial velocity behavior is examined for different values of prominent parameters. Figure 4a shows that by increasing the magnetic parameter, the axial velocity decreases near the axis of tube because the Lorentz’s force resists the fluid to flow in the centre of the tube due to magnetic field whereas fluid is at rest on the lateral surface of the tube. Figure 4b shows that increase in cilia length decreases the magnitude of the axial velocity at the centre of the tube while in the vicinity of the walls the axial velocity also decreases but in the opposite direction. From Fig. 4c, one can notice that magnitude of the axial velocity decreases in the centre of the tube by increasing the Jeffrey parameter i.e., the fluid becomes thick which retards the fluid flow in reverse direction. Figure 4d shows that by increasing the angle of inclination, magnitude of the axial velocity decreases at the centre of the tube but reverse trend is observed near the boundary walls, i.e., the inclined tube resists the flow in the reverse direction.

**Fig. 5:**
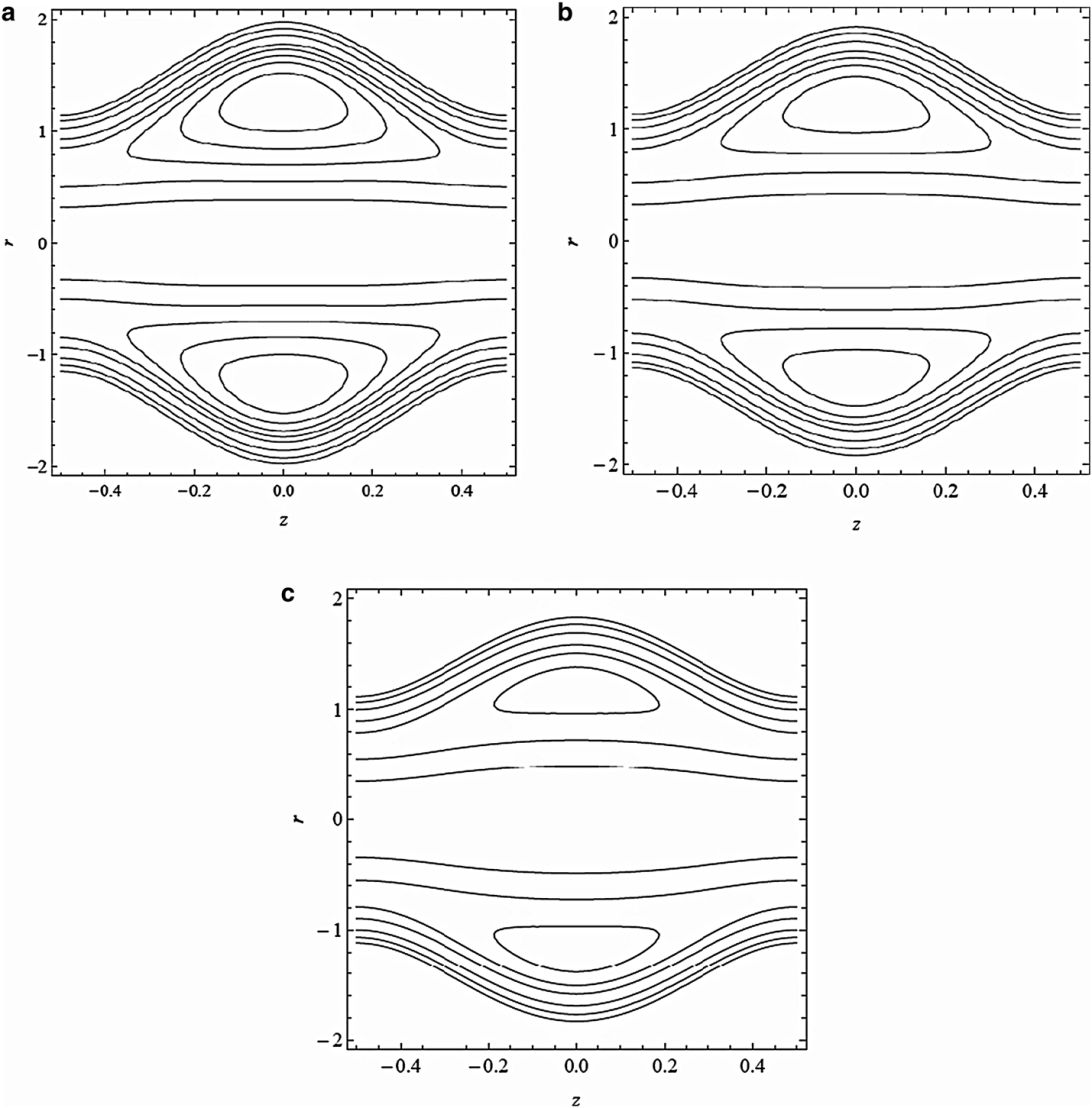
Streamline patterns for variation of *M* **a** *M* = 0.3, **b**  *M* = 0.9, **c**  *M* = 1.5 with $\epsilon =0.2, \alpha =0.4, \beta =0.4, \lambda _{1}=0.1, Fr=0.1, \textit{Re}=0.1, \theta =\frac{\pi }{6}$ and $\bar{Q}=3$

**Fig. 6:**
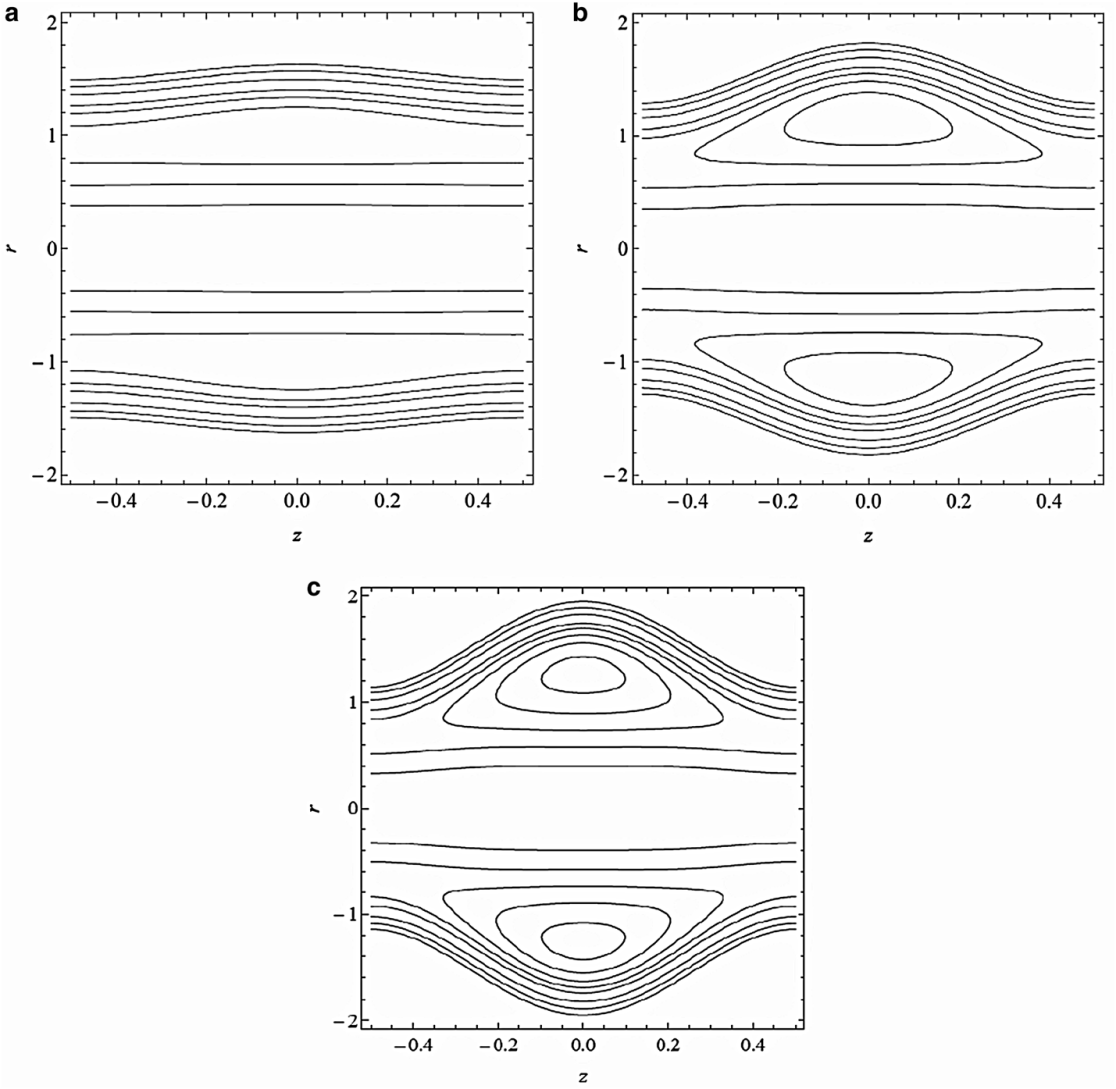
Streamline patterns for variation of cilia length parameter $\epsilon$ **a** $\epsilon =0.05$, **b** $\epsilon =0.16$, **c** $\epsilon =0.2$ with $M=0.5, \alpha =0.4, \beta =0.4, \lambda _{1}=0.1, Fr=0.1, \textit{Re}=0.1, \theta =\frac{\pi }{3}$ and $\bar{Q}=3$

**Fig. 7:**
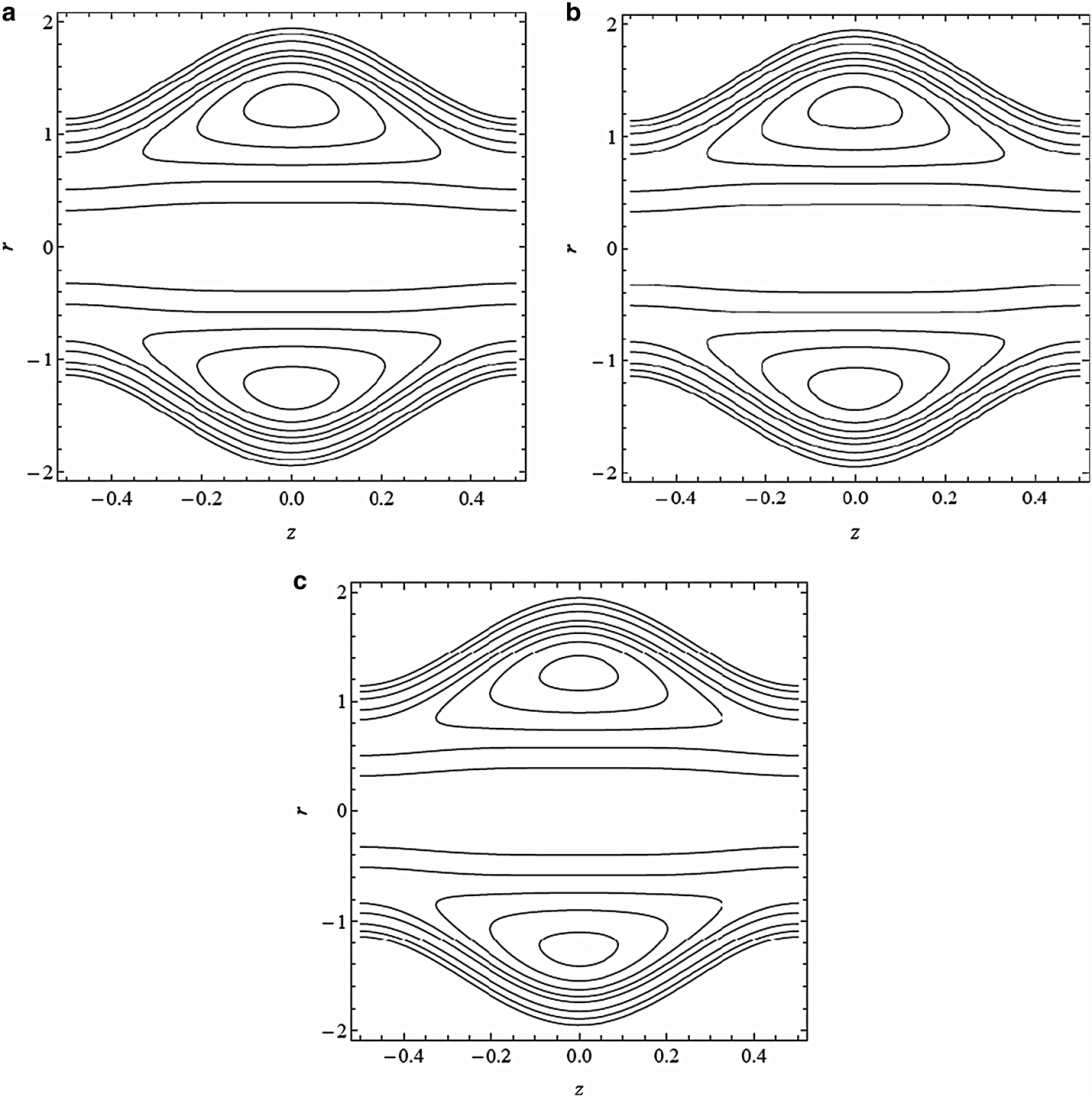
Streamline patterns for variation of angle of inclination *θ* **a** $\theta =0$, **b**  $\theta =\frac{\pi }{4}$, **c** $\theta =\frac{\pi }{2}$ with $M=0.5, \alpha =0.4, \beta =0.4, \lambda _{1}=0.1, \epsilon =0.2, Fr=0.1, \textit{Re}=0.1$, and $\bar{Q}=3$

Figure 5a–c show the graphs of streamlines for different values of Hartmann parameter *M*. It is observed that the size and circulation of the trapped bolus decrease with an increase in the value of *M*. It is further noted that the trapped bolus disappears for *M* = 1.5. This might be due to decelerating effect of magnetic force on the flow velocity. Figure 6a–c highlight that the size and circulation of the trapped boluses increase by increasing the cilia length parameter $\epsilon$ which indicates that circulation formed by the ciliary movement is dominant by increasing the cilia length. Figure 7a–c depict that the size of the bolus decreases with increase in the angle of inclination *θ* which shows that this parameter has significant effect on the viscosity of the the fluid.

## Conclusions

In this paper we have presented a theoretical approach to study ciliary motion of Jeffrey fluid in an inclined tube under the effect of magnetic field. The fluid flow is induced by the metachronal wave formed at cilia tip. The tube symmetry is produced by considering the metachronal wave on the cilia tip adjacent to fluid layer. We have adopted the long wavelength and low Reynolds number approximations that simplifies the problem. Cilia motion in Newtonian and non-Newtonian fluids is a current topic of interest. Different studies exit in literature in which the cilia induced flow in different geometries and stresses are discussed (Siddiqui et al. 2010, 2014a, [Bibr CR38][Bibr CR17]; Akbar et al. 2014, 2016). The exact analytical results obtained by Siddiqui et al. (2014b) can be captured as a limiting case (by taking *θ* and *λ*_1_ → 0) and are found to be in good agreement with the results of Siddiqui et al. (2014b). The effects of cilia length parameter $\epsilon$, Hartmann number *M*, Jeffrey parameter *λ*_1_ and angle of inclination *θ* on pressure rise Δ*p*, pressure gradient $\frac{dp}{dz}$, axial velocity *w* and stream function $\psi$ are investigated. It is observed that the amplitude of the pressure gradient increases with the increase in angle of inclination, Hartmann number and cilia length whereas the amplitude of $\frac{dp}{dz}$ decreases by increasing the Jeffrey parameter in the centre of the tube. Further the velocity profiles decrease by increasing the Hartmann number but reverse effect is observed by increasing the Jeffrey parameter.

## List of symbols

*U*, *W*: velocity components in the fixed frame
*u*, *w*: velocity components in the wave frame
*R*, *Z*: cylinderical polar coordinates in fixed frame
r, *z*: cylinderical polar coordinates in wave frame
*P*: pressure in fixed frame
*p*: pressure in wave frame
*t*: time
*f*, *g*: parametric representations of elliptical path
*a*: average radius of the tube
*c*: speed of metachronal wave
*g*: acceleration due to gravity
*J*: current density
*B*: total magnetic field
*M*: Hartmann number
*Re*: Reynolds number
*Fr*: Froude number
*Q*: volume flow rate
$\bar{{Q}}$: average volume flow rate
$$\frac{dp}{dz}$$: pressure gradient
Δ*p*: pressure rise
*α*: eccentricity of the elliptical path
*ρ*: fluid density
*μ*: viscosity
*λ*_1_, *λ*_2_: Jeffrey fluid parameters
*θ*: angle of inclination
$$\psi $$: stream function
$$\epsilon $$: cilia length
*λ*: wavelength of metachronal wave
*β*: ratio of *a* and *λ*
*σ*: electrical conductivity
